# Efficacy and safety of Shumian capsules in treating insomnia: A systematic review and meta-analysis

**DOI:** 10.1097/MD.0000000000028194

**Published:** 2021-12-17

**Authors:** Cuiying Wang, Yuying Yang, Xiao Ding, Jiamin Li, Xue Zhou, Jing Teng, Xianghua Qi

**Affiliations:** a Shandong University of Traditional Chinese Medicine, Jinan, China; b Tianjin University of Traditional Chinese Medicine, Tianjin, China; c Affiliated Hospital of Shandong University of Traditional Chinese Medicine, Jinan, Shandong, China; d Hunan University of Traditional Chinese Medicine, Changsha, China.

**Keywords:** efficacy and safety, insomnia, meta-analysis, Shumian capsules

## Abstract

**Background:**

It is known to all that the incidence of insomnia is becoming higher and higher with the increase of people's life stress. To some extent, it has brought about bad effects on people's life, work, study, and health, such as mental exhaustion, low work efficiency, and mood irritability. Now there are medications and non-medications methods for insomnia. As one of the treatments for insomnia, western medicine is to prolong the sleeping time and improve the anxious mood. However, taking western medicine to treat insomnia can also be accompanied by some adverse reactions at the same time, such as drug dependence, an allergic reaction, and so on. Traditional Chinese medicine therapy is based on syndrome differentiation and holistic concept. Shumian capsules (SM) are a kind of proprietary Chinese medicine for insomnia, which have the effect of relieving depression and calming the mind. But there are no studies on the efficacy and safety of SM in the treatment of insomnia. Therefore, I will provide a systematic review and meta-analysis to evaluate the efficacy and safety of SM for insomnia.

**Methods:**

All the studies searched were from PubMed, EMBASE, Web of Science, Cochrane Library, Chinese National Knowledge Infrastructure, and WanFang databases, and the studies types included in the analysis were all randomized controlled trials. All the retrieval contents were completed independently by 2 researchers, and a third reviewer would be involved when there existed any disagreement. The eligible studies were screened out according to the inclusion criteria and exclusion criteria, and some useful information was extracted and made into a feature table, including the year of the included studies, the age, and disease course of the participants in the studies and intervention methods, etc. Cochrane risk-of-bias tool was used to evaluate the quality of literature and meta-analysis was conducted by RevMan 5.4 software.

**Results:**

A total of 9 articles including 709 participants were included in the study after screening out. The primary outcomes of statistical analysis were cure rate and total effective rate, while the secondary outcomes included Pittsburgh sleep quality index score and incidence of adverse reactions. The results showed that Pittsburgh sleep quality index score of the SM group and Western medicine group were statistically significant (MD = –0.50, 95% confidence interval [CI] = [–0.78, –0.22], *P* = .0005). The total effective rate of the SM group was slightly higher than that of the Western medicine group, but there was no statistical significance (relative risk [RR] = 1.03, 95% CI = [0.95,1.13], *P* = .43).

**Conclusion:**

This meta-analysis provides evidence for the efficacy and safety of SM in the treatment of insomnia, and provides a new idea for the clinical treatment of insomnia. But more research is needed to support further evidence.

## 1 Introduction

Insomnia is a general sleep disorder in modern society, which has been considered to be a common risk factor for psychological and physical diseases.^[1]^ The prevalence of insomnia in the general population is 10% to 15%.^[2]^ In recent years, due to the acceleration of people's pace of life, the incidence rate of insomnia has increased year by year. The National Sleep Foundation's Sleep Timing Panel recommends that adults get 7 to 9 hours of sleep a night (7–8 hours for seniors).^[3]^ Studies have shown that older people, women, and people with high level of stress are more likely to develop the disease. In addition, taking certain medications can also cause insomnia, such as glucocorticoids and non-steroidal anti-inflammatory drugs.^[4]^ Moreover, insomnia is not only a common risk factor for certain diseases, but also a concomitant symptom of certain diseases, including but not limited to coronary heart disease, diabetes, hypertension, etc. Insomnia is a kind of disease characterized by frequent failure to get normal sleep, mainly manifested as insufficient sleep time and depth, including difficulty falling asleep, easy to wake up from sleep, difficulty falling asleep again after waking up, and in severe cases, sleepless all night.^[4,5]^ Western medicine for insomnia treatment is mainly divided into psychotherapy and drug therapy. Psychotherapy mainly includes simple insomnia behavior therapy and cognitive insomnia behavior therapy. In addition, drug therapy mainly includes benzodiazepines, non-benzodiazepines, melatonin receptor agonists, serotonin-receptor antagonists, and hypnotic antidepressants.^[4]^ However, drug dependence, withdrawal syndrome, and other adverse effects have made the clinical treatment of insomnia more difficult. Besides, medications for insomnia can also lead to behavioral and cognitive changes. Based on the disadvantages of these treatments, we should find more effective treatments to help people solve their sleep problems.^[6]^

With the development of social science and technology and the deepening of people's understanding of diseases, traditional Chinese medicine (TCM) has attracted more and more people's attention. In fact, TCM has played a very effective role in the treatment of various diseases. Treatment based on syndrome differentiation and holistic concept are important components of the basic theory of TCM.^[7]^ In the treatment of insomnia, the application of TCM therapy mainly includes acupuncture, tuina, Chinese herbal medicine, etc.^[8–11]^ There are many other TCM non-drug therapies which are also widely used, such as TCM psychotherapy, aromatherapy, music therapy, Qigong, Wuqinxi, and so on.^[12]^ Thus it can be seen that various TCM treatment methods are widely used in the treatment of improving patients’ clinical symptoms.

According to the continuous improvement of traditional Chinese medicine dosage forms and the improvement of traditional Chinese medicine technical level, many traditional Chinese patent medicine preparations such as Shumian capsules (SM) are widely used in clinical practice. SM is mainly composed of Huangqin (Bupleurum), Suanzaoren (jujube seed), Hehuanhua (acanthopanax), Baishao (white peony), Hehuanpi (albizia bark), Jiangcan (silkworm), Chantui (Cicada slough), and Dengxincao (rush herb). SM is a common drug for insomnia in China, and there have been many randomized controlled clinical trials to support this theory. However, the efficacy of SM in the treatment of insomnia has not been systematically evaluated. Therefore, our study will evaluate and analyze the safety and efficacy.

## 2 Methods

### 2.1 Study registration

This systematic review and meta-analysis has been registered on PROSPERO platform with an assigned registration number CRD42020214280, basing on the Preferred Reporting Items for Systematic Reviews and Meta-Analyses Protocols statement guidelines.

### 2.2 Eligibility criteria

#### 2.2.1 Types of studies

Only randomized controlled trials (RCTs) were included in the study. Other studies, such as those that are not RCTs, case reports, or other types of studies, were not included.

#### 2.2.2 Types of participants

Participants were required to have a definite diagnosis of insomnia consistent with ICSD-3 in the International Classification of Sleep Disorders II or The Chinese Guidelines for the Diagnosis and Treatment of Insomnia. In addition, the age, sex, and course of disease of the patients involved in the study were not restricted.

#### 2.2.3 Type of interventions

The experimental group was given SM alone or combined with western medicine or other conventional treatment, while the control group was given placebo or western medicine alone. If the experimental group received conventional treatment, the control group must receive the same conventional treatment alone.

#### 2.2.4 Types of outcome measurements

##### 2.2.4.1 Primary outcomes

The primary outcomes include clinical effective rate and cure rate. The clinical effective rate was calculated according to the Guiding Principles of Clinical Research on New Drugs of Traditional Chinese Medicine.^[13]^ The criteria for clinical efficacy scores are as follows: cure: clinical symptoms disappear, >6 hours of sleep at night, good sleep quality, and energetic after waking; significantly effective: the clinical symptoms were significantly improved, and the night sleep time was increased by >3 hours; effective: the clinical symptoms were improved and the night sleep time was increased <3 hours; invalid: no improvement in clinical symptoms and no increase in sleep time. Total effective rate = (cure + significantly effective + effective)/total number of cases × 100%, cure rate = cure/total number of cases × 100%.

##### 2.2.4.2 Secondary outcomes

The secondary outcomes mainly included Pittsburgh sleep quality index (PSQI) score and incidence of adverse reactions. The PSQI is composed of 19 self-rated questions and 5 questions rated by sleep companion. Only 19 self-rated questions were scored. The 19 self-rated questions consist of 7 factors with a score of 0 to 3. The cumulative score of each factor component is the total score of the PSQI, which ranges from 0 to 21.^[14]^ The higher the score, the worse the sleep quality. The adverse reactions include dizziness, headache, fatigue, gastrointestinal reactions, and memory loss, etc.

### 2.3 Study search

#### 2.3.1 Electronic searches

All the studies searched were from PubMed, EMBASE, Web of Science, Cochrane Library, Chinese National Knowledge Infrastructure, and WanFang databases from their inception to October 2020.

#### 2.3.2 Searching other resources

Other sources of research include conference papers, journal references, and papers not available in electronic editions.

#### 2.3.3 Search strategy

The keywords we search for are: (“Shumian capsules” OR “SM”) AND (“insomnia” OR “sleep disorder”) AND (“randomized controlled trial” OR “randomized”).

### 2.4 Data collection and analysis

#### 2.4.1 Selection of studies

**Figure 1:**
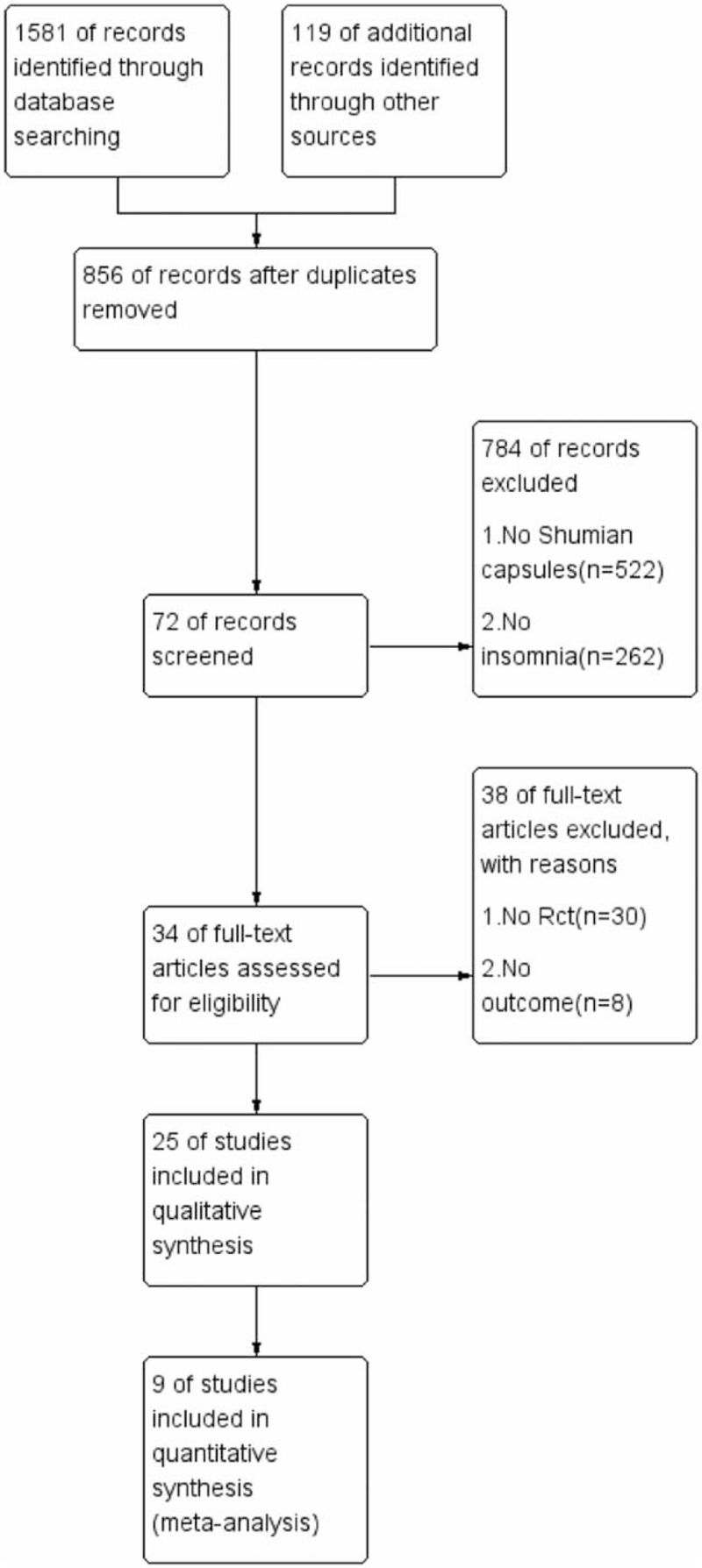
Flow chart of study selection.

Two reviewers will independently collect qualified studies according to the search strategy. All searched articles are placed in the literature management software Endnote (versionX9, Thomson Reuters). Then 2 reviewers independently screened and evaluated the title and abstract of the paper according to the inclusion criteria and exclusion criteria, and eliminated the unqualified studies. Two reviewers extracted data from qualified studies and checked each other. Any disagreement in the process of screening articles and extracting data shall be decided by 2 reviewers through consultation. If no agreement can be reached, a third party shall enter into the discussion and negotiate. The process of studies selection is presented in a preferred reporting project flow chart for systems review and meta-analysis (PRISMA) (Fig. 1).

#### 2.4.2 Data extraction and management

All data extraction shall be completed independently by 2 reviewers. In case of any disagreement, 2 reviewers shall negotiate and resolve it. If no agreement can be reached, a third party shall participate in the discussion. The data were extracted as follows: author, sample size, average age, average course of disease, intervention plan, main outcome, duration of treatment, and adverse reactions.

#### 2.4.3 Risk of bias assessment

The 2 researchers independently assessed the methodological treatment included in the study using the Cochrane Manual's “Risk of Bias Assessment Tool.” The quality of the included literature will be assessed according to the following methods: random sequence generation (selection bias), allocation concealment (selection bias), blinding of participants and personnel (performance bias), blinding of outcome assessment (detection bias), incomplete outcome data (attrition bias), selective reporting (reporting bias), and others bias. Each item is classified as “Low risk,” “High risk,” or “Unclear risk.”^[15]^

#### 2.4.4 Data synthesis and analysis

The data analysis will be performed by Review Manager 5.4 software from Cochrane Collaboration (RevMan, version 5.4, the Nordic Cochrane Center, the Cochrane Collaboration). For binary categorical variables, relative risk (RR) and 95% confidence interval (CI) are given. For continuous variables, the mean difference and 95% CI are given. According to the results of the heterogeneity test, the effect model was selected. If *I*^2^ > 50%, the random effect model was selected. If *I*^2^ < 50%, the fixed effect model is selected. Funnel plots were used to assess study publication bias, Funnel plan is used to evaluate the publication bias of the study. If the 2 sides are symmetrical, there is no obvious publication bias. If the 2 sides are asymmetric, it indicates that there may be publication bias.

### 2.5 Ethic approval

Ethical approval and patient informed consent are not required for this study, because we only extract relevant data from published articles. The results of this systematic review will be published in peer-reviewed journals. It provides sufficient evidence-based medical support for the clinical treatment of insomnia.

## 3 Results

### 3.1 Search results

At the beginning, the 2 reviewers searched a total of 1700 studies, and 856 articles were left after the removal of duplicate literatures. Then 784 articles were excluded except those that did not involve SM and insomnia. In addition, there were 38 articles that did not involve RCTs and had no major results. Finally, after reading through the full text of the article, 9 studies that meet the inclusion criteria are selected.^[16–24]^ The flow chart of the literature screening process is shown in Fig. 1.

### 3.2 General characteristics of included studies

A total of 709 patients were included in the study, including 353 in the treatment group and 356 in the control group. In these 9 studies, all the treatment groups were treated with SM alone, while in the control group, 1 study was treated with alprazolam, 1 study with diazepam, and the remaining 7 studies were treated with estazolam. The duration of treatment ranged from 0.25 to 6 weeks. In addition, all the 9 studies showed adverse reactions of varying degrees.

### 3.3 Quality evaluation

**Figure 2:**
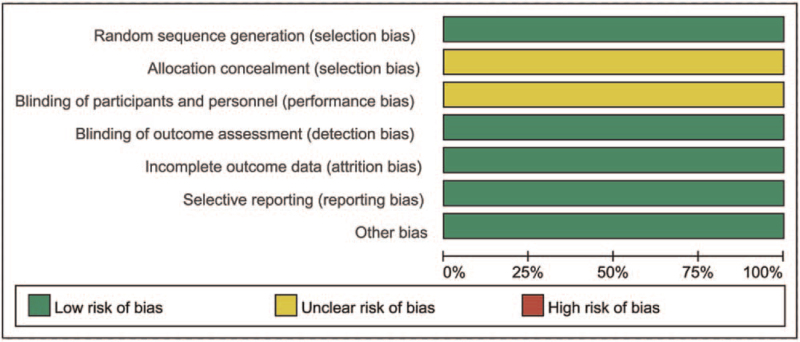
Risk of bias graph.

**Figure 3:**
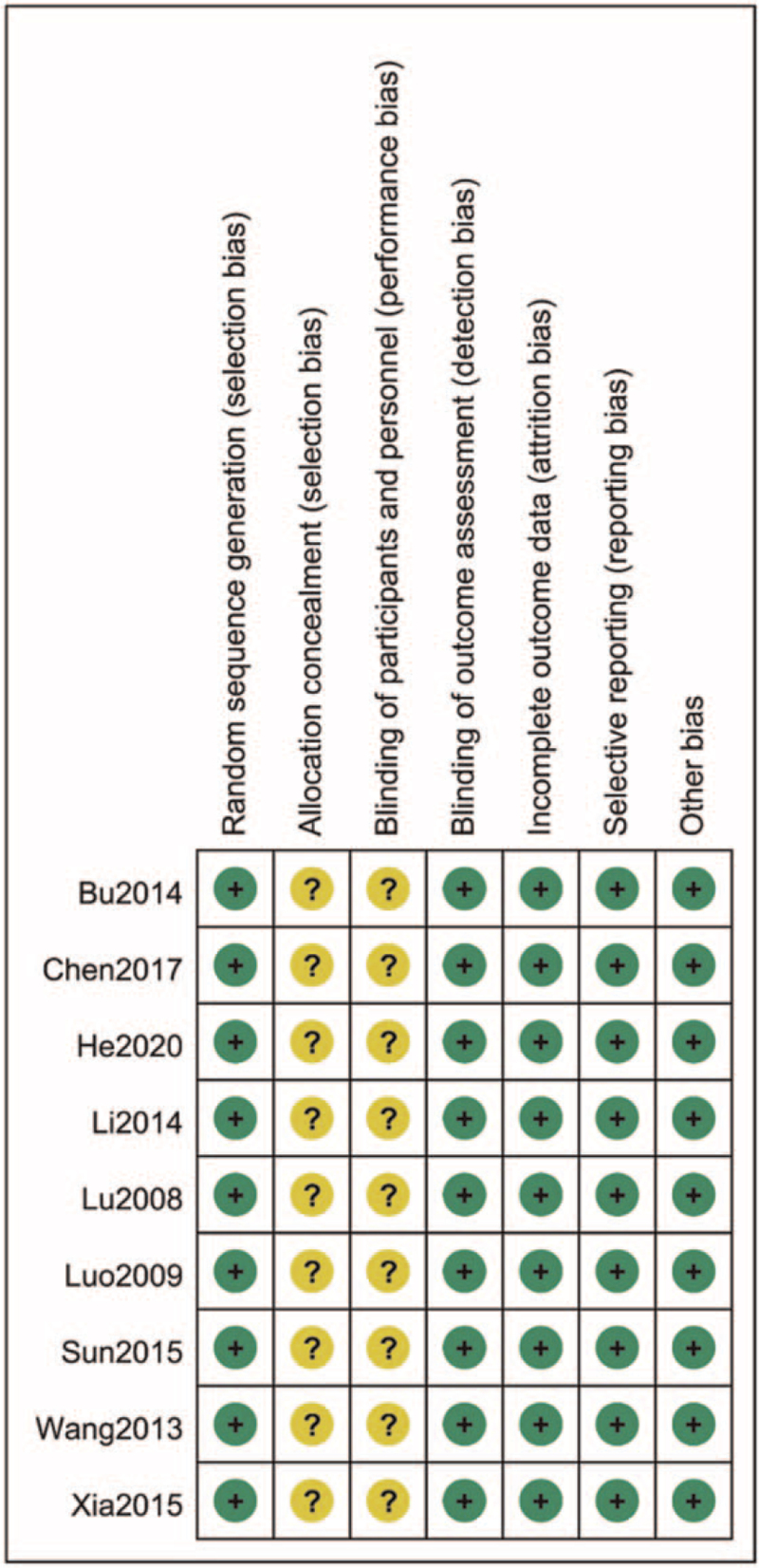
Risk of bias summary.

All the studies mentioned the application of random allocation, but did not mention the method of random classification. Allocation concealment and double blindness were not mentioned in any of the studies. All the study data were complete, and there was no bias in detection and selection of reports. No other risk of bias was found. According to the Cochrane Risk of Bias Tool, the quality of the articles selected was moderate. The risk of bias was presented in Figs. 2 and 3.

## 4 Meta-analysis results

### 4.1 Clinical efficacy

**Figure 4:**
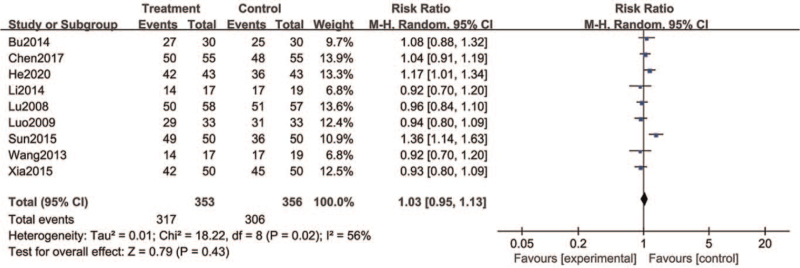
Forest plots of the clinical effective rate of SM compared with western medicine. SM = Shumian capsules.

The curative effect is evaluated according to the Guiding Principles of Clinical Research on New Drugs of Traditional Chinese Medicine, which is divided into cure, obvious effect, effective, and ineffective. According to the results of the heterogeneity test (*I*^2^ = 56%), the random effects model was selected. The results showed that the clinical effective rate of the treatment group (89.8%) was higher than that of the control group (85.9%). However, there was no statistical significance between the 2 groups (RR = 1.03, 95% CI = [0.95, 1.13], *P* = .43) (Fig. 4).

The source of heterogeneity was examined by excluding references one by one, and the results showed that after the elimination of Sun and Jin,^[22]^ the *I*^2^ value was 14%, while after the elimination of the remaining 8 articles, the *I*^2^ value was still >50%.

### 4.2 Cure rate

**Figure 5:**
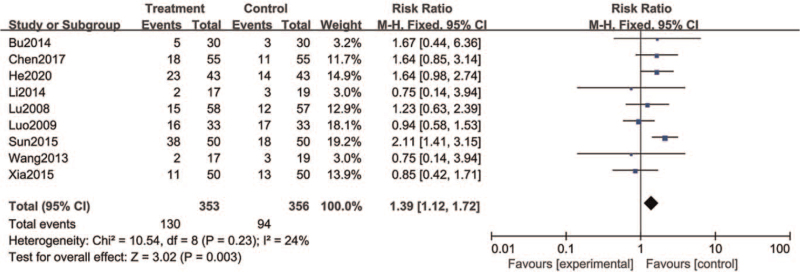
Forest plots of the clinical cure rate of SM compared with western medicine. SM = Shumian capsules.

According to the results of heterogeneity test (*I*^2^ = 24%), the effect model was selected as fixed effect model. The results showed that the cure rate of the treatment group (36.8%) was higher than that of the control group (26.4%). There was a significant statistical difference between the 2 groups (RR = 1.39, 95% CI = [1.12, 1.72], *P* = .003) (Fig. 5).

### 4.3 Adverse reactions

**Figure 6:**
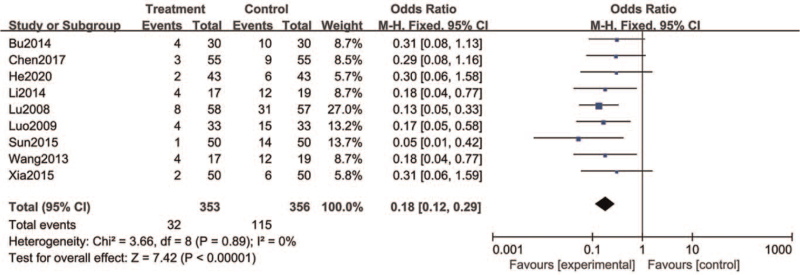
Forest plots of incidence of adverse reactions.

Adverse events, including but not limited to headache, dizziness, fatigue, gastrointestinal reactions, were reported in all the included literatures. There were 32 cases of adverse reactions in the treatment group and 115 cases in the control group. The results showed that there was a significant statistical difference between the treatment group and the control group (RR = 0.18, 95% CI = [0.12, 0.29], *P* < .00001) (Fig. 6).

### 4.4 PSQI score

The PSQI was used to assess patients’ sleep quality. The evaluation of clinical efficacy is based on PSQI reduction rate. The efficacy evaluation criteria is as follows: cure: 75% ≤ PSQI reduction rate ≤ 100%, significant effect: 50% ≤ PSQI reduction rate < 75%, effective: 25% ≤ PSQI reduction rate < 50%; Invalid: PSQI reduction rate < 25%. Reduction rate = ([total score before treatment − total score after treatment]/total score before treatment) × 100%. Only one article mentioned PSQI reduction rate, so it did not make and analyze forest plots.

### 4.5 Publication bias

**Figure 7:**
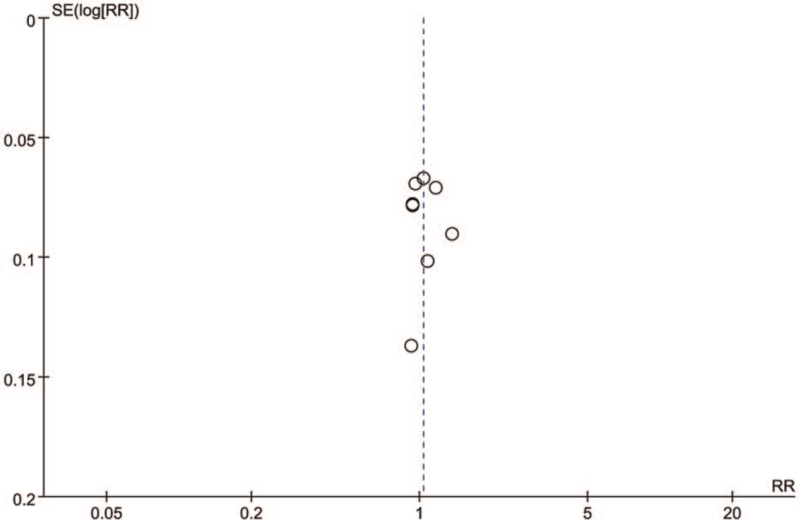
Funnel plot analysis of 9 trials for the outcomes.

Funnel plot was used to evaluate publication bias. If both sides of funnel plot are symmetric, it indicates that there is no obvious publication bias, but if both sides are asymmetric, it indicates that there may be publication bias. Figure 7 is the evaluation of publication bias for the 9 articles included in this study. We can see that there is no obvious asymmetry in the shape of funnel plot, indicating that there is no significant publication bias in this study.

## 5 Discussion

This meta-analysis was used to evaluate the efficacy and safety of SM in the treatment of insomnia. The main outcomes used to evaluate clinical efficacy included clinical effective rate, cure rate, incidence of adverse reactions, and PSQI score. According to the results, the clinical effective rate of the treatment group was slightly higher than that of the control group, but there was no statistical difference between the 2 groups, indicating that compared with western medicine, SM did not significantly improve the clinical efficacy in the treatment of insomnia. It is worth noting that the adverse reaction events caused by SM were far less than those caused by western medicine, with a significant statistical difference (*P* < .00001). Therefore, SM are safe and effective in treating insomnia.

Traditional Chinese medicine (TCM) has its unique advantages in treating insomnia, and has definite curative effect in improving clinical symptoms. Syndrome differentiation is one of the characteristics of disease differentiation and treatment in traditional Chinese medicine. A syndrome is a generalization of the nature of a certain stage or type of disease in the course of a disease. Traditional Chinese medicine (TCM) believes that emotional injury is the main cause of insomnia. SM are mainly used for the insomnia of liver-depression type, and have the effect of soothing the liver and relieving depression, calming the heart and calming the nerves. Jujube seed, white peony root, and silkworm in SM are rich in tryptophan (which is the precursor of 5-HT). Therefore, taking SM can supplement the deficiency of 5-HT. Bupleurum in Shuman capsules can regulate the function of sympathetic nerve and parasympathetic nerve, and improve the content of norepinephrine, so as to regulate slow wave sleep and fast wave sleep together with the locus amoeba. Through the 2 functions, the purpose of improving the sleep quality and restoring independent sleep can be achieved together, so as to achieve the effect of curing insomnia.^[25]^

This study has the following limitations. The analysis of clinical efficiency has certain heterogeneity, and the source of heterogeneity, namely Sun and Jin,^[22]^ is found through the method of excluding literatures article by article. After reading the whole study, the heterogeneity may be caused by reasons such as small sample size and lack of quality control. In addition, although all the literatures included in this study mentioned random grouping, they did not mention the method of random allocation, allocation concealment, or double-blindness. In addition, none of the studies mentioned follow-up, making it impossible to assess the long-term efficacy of SM. Despite the above limitations, this study provides useful clinical value to a certain extent. In the future, it is hoped that a multicountry, multicenter, large-sample randomized double-blind controlled trial can be conducted.

## 6 Conclusion

To sum up, SM is an effective and safe means to treat insomnia, especially with the advantage of less adverse reactions.

## Author contributions

**Conceptualization:** Cuiying Wang.

**Data curation:** Yuying Yang, Xiao Ding, Xue Zhou.

**Formal analysis:** Cuiying Wang, Yuying Yang, Jiamin Li.

**Methodology:** Jing Teng, Xianghua Qi.

**Project administration:** Xianghua Qi.

**Supervision:** Xianghua Qi.

**Validation:** Cuiying Wang.

**Writing – original draft:** Cuiying Wang.
